# Response times in Ecological Momentary Assessment (EMA): shedding light on the response process with a drift diffusion model

**DOI:** 10.1007/s12144-023-04773-0

**Published:** 2023-05-27

**Authors:** Stefan Schneider, Raymond Hernandez, Doerte U. Junghaenel, Bart Orriens, Pey-Jiuan Lee, Arthur A. Stone

**Affiliations:** 1 Center for Self-Report Science & Center for Economic and Social Research, University of Southern California, 635 Downey Way, Los Angeles, CA 90089-3332, USA; 2 Department of Psychology, University of Southern California, Los Angeles, CA, USA; 3 Leonard Davis School of Gerontology, University of Southern California, Los Angeles, CA, USA; 4 Center for Economic and Social Research, University of Southern California, Los Angeles, CA, USA

**Keywords:** Ecological Momentary Assessment, Response times, Affect, Drift diffusion model, Response processes

## Abstract

Mental processes underlying people’s responses to Ecological Momentary Assessments (EMA) have rarely been studied. In cognitive psychology, one of the most popular and successful mental process models is the *drift diffusion model*. It decomposes response time (RT) data to distinguish how *fast* information is accessed and processed (“drift rate”), and how *much* information is accessed and processed (“boundary separation”). We examined whether the drift diffusion model could be successfully applied to people’s RTs for EMA questions and could shed light on between- and within-person variation in the mental process components underlying momentary reports. We analyzed EMA data (up to 6 momentary surveys/day for one week) from 954 participants in the Understanding America Study (29,067 completed measurement occasions). An item-response-theory diffusion model was applied to RTs associated with 5 momentary negative affect ratings. As hypothesized, both diffusion model parameters showed moderate stability across EMA measurement occasions. Drift rate and boundary separation together explained a majority of the variance in the observed RTs and demonstrated correspondence across different sets of EMA items, both within and between individuals. The parameters related in theoretically expected ways to within-person changes in activities (momentary work and recreation) and person-level characteristics (neuroticism and depression). Drift rate increased and boundary separation decreased over the study, suggesting that practice effects in EMA consist of multiple distinctive cognitive processes. The results support the reliability and validity of the diffusion model parameters derived from EMA and provide initial evidence that the model may enhance understanding of process underlying EMA affect ratings.

## Introduction

Interest in the assessment of how people feel, think, and act in their natural daily environments has risen dramatically in psychological research in recent decades. Chief among methods to capture people’s experiences in real time is ecological momentary assessment (EMA; Stone & Shiffman, 1994). Using EMA, individuals are prompted several times per day over multiple days to collect momentary self-reports repeatedly and with a temporal granularity that traditional recall questionnaires cannot afford. This provides many opportunities to study the temporal dynamics of experiences (e.g., how individuals regulate their emotions) (Carstensen et al., 2011; Schneider et al., 2020), and, because respondents are reporting on their immediate experiences, reduces biases associated with memory heuristics.

EMA is often considered a gold standard of self-report assessment (Kahneman et al., 2004; Schneider & Stone, 2016; Stone et al., 2023). However, how people answer questions in EMA is an understudied topic. To answer momentary questions, participants need to access the relevant information (e.g., about their current emotions) from working memory, process the information, and make decisions about the best possible answer, repeatedly for multiple questions, and under real life circumstances. A better understanding of these processes could advance our knowledge about the internal workings of EMA and about the ebb and flow of information processing in real life.

Response times (RTs) to EMA questions represent one source of information to potentially elucidate the mental processes underlying EMA responses. The collection of RTs has been one of the most important means for investigating hypotheses about people’s information processing in many areas of social science (van Zandt, 2002). RTs have been used as indicators of processing speed (“mental chronometry”) (Meyer et al., 1988) and neurological functioning (Forstmann et al., 2010), to inform ability measurement in cognitive and educational testing (Kyllonen & Zu, 2016), to improve survey methodology (Bassili & Scott, 1996), and to test psychological theories about attitudes and emotions (Fazio, 1990; Robinson & Clore, 2002). The dramatic increase in the use of electronic devices (e.g., smartphones) for momentary self-report data capture in recent years has made RTs widely available in contemporary EMA research (Pejovic et al., 2016; Trull & Ebner-Priemer, 2013). To date, we are only beginning to understand the mental processes underlying peoples’ EMA evaluations of their everyday experiences (Stone et al., in press). However, several studies have demonstrated the value of using RTs in EMA, as evident in the following literature review.

### Use of response times to capture mental processes in EMA

In one of the few lines of research in this area, the extent to which individuals are aware of their emotions (affective clarity), was thought to be indexed by RTs to momentary affect questions (Lischetzke et al., 2005). Lischetzke et al. (2005) asserted that the greater an individual’s momentary affective clarity, the more easily assessible their emotional experiences should be at a given moment and the less time should be needed to provide a rating of momentary affect. The indirect assessment of affective clarity via RTs parallels the measurement of attitude strength in attitude research, where faster RTs have been associated with stronger and more immediately accessible attitudes – that is, those that come to mind quickly (Bassili, 1996; Fazio, 1990). Indeed, studies have shown that RTs for momentary mood ratings were associated with momentary self-reports of affect clarity, supporting convergent validity of RTs as affective clarity measure (Lischetzke et al., 2005, 2011). Faster affect ratings in EMA have also been associated with more positive daily emotion regulation strategies and better mental health (Arndt et al., 2018; Lischetzke et al., 2005, 2011). For example, people with higher neuroticism and depression levels were found to show slower RTs when responding to momentary negative affect items, in line with the hypothesis that neuroticism and depression are associated with less efficient emotional information processing and lower accessibility of current negative affective states (Thompson et al., 2015).

A second line of research has collected RTs to evaluate the *quality* of responses in EMA. In this research, fast responses to EMA questions are viewed as an indicator of cursory information processing and potential measurement biases. In view of long-standing concerns that intensively repeated assessments with EMA may lead to measurement reactivity and induce shifts in responding over time, Arslan et al. (2021) examined whether RTs on repeated mood and symptom ratings changed over a period of up to 70 days. RTs became increasingly fast over the course of the study and with repeated assessments (Arslan et al., 2021), in line with potential reactive arrangements. Similarly, exceedingly fast RTs have been argued to be an indication of survey satisficing or careless responding in EMA (Jaso et al., 2022). Corresponding with traditional survey research contexts, where RTs have been used as an index of careless responding for many years (Meade & Craig, 2012), fast RTs may flag invalid responses due to insufficient effort and limited mental processing of EMA items.

Despite the increasing recognition of the potential utility of collecting RT data in EMA research, there is considerable ambiguity about the exact mental processes captured by these RT data. As the research summary above illustrates, respondents may give fast answers to momentary self-report questions because they were able to access the relevant information swiftly and efficiently (i.e., high emotional clarity), or because they put little effort in their responses and only engaged in a cursory search for the information (i.e., survey satisficing). Conversely, people can be slower to respond because the requisite information was not readily mentally accessible, or because they responded with great caution. Clearly, RTs comprise several cognitive-motivational processes and components that are confounded when observed RTs are taken at face value.

### A drift diffusion model to disambiguate response times in EMA

**Fig. 1:**
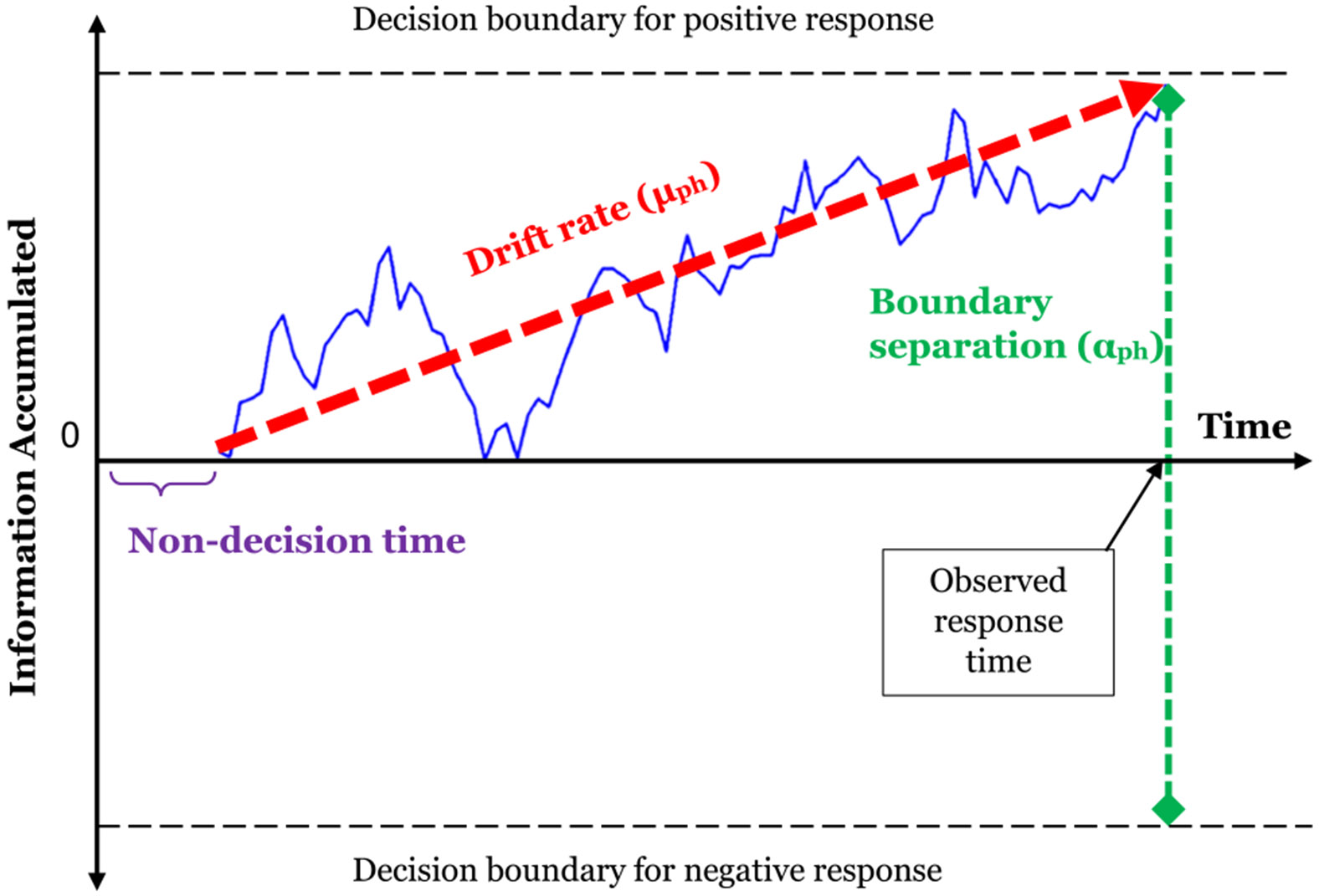
Schematic illustration of the decision process in a drift diffusion model

To help overcome these ambiguities and gain greater insight into the cognitive processes inherent in the speed of responding to EMA questions, we turn to mathematical models of RTs from cognitive psychology. These models were developed explicitly with the intention to decompose observed RTs into more interpretable and theoretically plausible mental process parameters (Kyllonen & Zu, 2016). One of the most popular and successful process models for analyzing RT data is the *drift diffusion model* (Ratcliff, 1978; Ratcliff & Rouder, 1998). The drift diffusion model belongs to the class of random walk models that are meant to explain simple-choice decision problems in which a respondent needs to choose between two alternative response options (see Fig. 1). The decision process is viewed as a continuous, but noisy, information search process in which the respondent accumulates accessible information relevant for the decision over time (the jagged line in Fig. 1). Following a period of nondecision time, the respondent accumulates information until the total amount of information accrued reaches the upper or lower boundary, resulting in a positive (upper boundary reached) or negative (lower boundary reached) response.

The two central parameters of the diffusion model are the drift rate and boundary separation parameters (see Fig. 1). The *drift rate* (μ) is the mean rate (or speed) with which people process and accumulate relevant information to reach one of the boundaries. A high drift rate is interpreted as meaning that information in favor of one of the response choices is highly accessible and accumulates quickly, with little evidence coming to mind favoring the alternative. As discussed above, this process is assumed to underly emotional clarity. The higher the drift rate, the steeper the (positive or negative) slope of μ, whereby, all else held equal, a higher drift rate results in a faster observed RT.

The second parameter, called *boundary separation* (α), is the distance between the upper and lower boundary and it is meant to represent the amount of evidence that the person accumulates before executing a response. The boundary separation parameter is interpreted as *response caution*. It reflects the idea that individuals rarely retrieve all information that may be relevant for a decision, but truncate the search process as soon as “sufficient” information has accrued to give a response with enough subjective certainty. Boundary separation is essentially related to the widely recognized speed-accuracy tradeoff (Kyllonen & Zu, 2016). A narrower boundary separation leads to faster, yet less precise and likely less accurate answers because relatively less information is retrieved, the process presumed to underly survey satisficing and careless responding discussed above. By contrast, a wider boundary separation leads to slow RTs, yet more thorough and accurate answers (Kyllonen & Zu, 2016; Ratcliff et al., 2016).

In sum, drift rate and boundary separation parameters divide the observed RTs into two fundamental unobserved components capturing how *fast* information is accessed and processed (drift rate), and how *much* information is accessed and processed (boundary separation). In the present study, we examined whether the drift diffusion model can be successfully applied to RTs for EMA questions to disentangle components of information processing as people answer momentary questions about their experiences in daily life.

### The present study

The parameters of the diffusion model have been extensively validated in experimental studies (Matzke & Wagenmakers, 2009; Ratcliff et al., 2016) and the model has been used across various areas of psychological research, including research on neuropsychology (Zhang & Rowe, 2014), cognitive functioning (Schmiedek et al., 2007), and social and personality psychology (Lin et al., 2020). However, these applications of the diffusion model have almost exclusively focused on RTs obtained in speeded experimental tasks, such as lexical decision, brightness discrimination, letter identification, spatial decision, recognition memory, and signal detection tasks (Matzke & Wagenmakers, 2009).

There are many differences between typical diffusion model applications and its application to EMA, and these are summarized in Table 1. Whereas typical applications often involve many homogeneous repeated trials in which participants are explicitly asked for fast decisions under controlled laboratory conditions, EMA involves responding to relatively few different self-report items at a participant’s own pace in naturalistic settings. Importantly, most diffusion model applications have been based on response times collected in a single session (or few sessions), whereas EMA involves repeated measurements multiple times per day over multiple days. Accordingly, applying the diffusion model to EMA provides potentially unique opportunities to investigate both stable between-person differences as well as dynamic within-person variability in people’s information processing in everyday life.

To date, there have been no investigations of the extent to which the drift diffusion model can be successfully applied to RTs in EMA for the purpose of distinguishing the speed and amount of information processing underlying momentary self-reports. Accordingly, the goal of the present research was to provide a first examination of the reliability and validity of drift diffusion model parameters derived from RTs in EMA. Our study focuses on EMA reports of momentary negative affect, given that emotional experiences are very commonly examined in EMA studies, and given that RTs for negative affect items have been used as indirect measures of emotional clarity in previous EMA studies (Lischetzke et al., 2011; Thompson et al., 2015).

Our first research question was whether a diffusion model can capture systematic between-person and within-person (across EMA measurement occasions) sources of RT variability when applied to affect scales used in EMA. This question is especially important given that the number of EMA items administered per scale at each prompt is often limited. For example, the negative affect measure used in the current study consisted of only 5 items at each EMA assessment, which is substantially fewer items compared to common laboratory-based diffusion model applications which may include 100 decision making trials or more (Ratcliff et al., 2016). Thus, we asked whether and to what extent the diffusion model parameters (drift rate and boundary separation) can be reliably distinguished from each other when extracted from RTs for EMA affect ratings.

We addressed this question in three ways. The first way involved examining the test–retest stability of drift rate and boundary separation parameters across EMA measurement occasions. This was accomplished by comparing the between- and within-person variance components in the diffusion model parameters. Prior EMA research has documented that observed RTs show moderate stability across EMA prompts, with about 1/3 of the variance being between and 2/3 within-person (i.e., an intraclass correlation of about 0.3)(Thompson et al., 2015). If the diffusion parameters can be reliably captured in brief EMA affect scales, we would similarly expect moderate test–retest stabilities for drift rate and boundary separation parameters derived from individual EMA prompts.

#### Hypothesis 1

Drift rate and boundary separation parameters derived from RTs for EMA affect ratings demonstrate moderate stability across measurement occasions.

Additionally, if drift rate and boundary separation are distinguishable mental process components that give rise to the observed RTs in EMA, then we expect that the two diffusion parameters predict the observed RTs in opposite directions and together explain the large majority of variance in observed RTs, both at the between-person and at the within-person level. On both levels, we expected that faster RTs would be uniquely associated with higher drift rates (faster affect information processing; a positive association) and with lower boundary separation (lower response caution; a negative association).

#### Hypothesis 2

Drift rate and boundary separation parameters relate to observed RTs in opposite directions and together account for most of the RT variance within and between individuals.

Moreover, we expected the diffusion parameters derived from EMA negative affect items to show correspondence with the same parameters derived from a set of items with different content in the same EMA survey; we used items asking about stressful events for this purpose. That is, both drift rate and boundary separation should correlate moderately to highly with the same parameter derived from negative affect and stressful events items, whereas drift rate and boundary separation parameters should be only weakly correlated with each other (within and across the two sets of EMA items). We expected this pattern at the between-person and at the within-person level.

#### Hypothesis 3

Drift rate and boundary separation parameters calculated from different sets of EMA items show moderate to high correspondence within and between individuals.

Our second research question addressed the construct validity of the drift diffusion parameters. The drift diffusion model would be most valuable if its parameters had the potential to uncover theoretically expected patterns of relationships with cognitive processes underlying EMA responses that are confounded when observed RTs are taken at face value. To address this, we strategically selected a set of predictor variables for which we hypothesized that they could plausibly predict both *faster* and *slower* RTs at the same time. This pattern would occur whenever a situational or person characteristic is simultaneously associated with a higher drift rate (indirectly predicting faster RTs) and a higher boundary separation (indirectly predicting slower RTs), or vice versa. Since these effects work in opposite directions, their combination would result in effects on observed RTs that are weak or fully canceled out (MacKinnon et al., 2007).

On the within-person level, we theorized that this would be naturally the case when comparing RTs for EMA items completed across different momentary activities such as working and relaxing/regenerating. When individuals are mentally occupied with work activities, this creates competing demands that may require divided attention when completing EMA affect items. In view of limited mental resources, divided attention decreases the speed of mental processes and reduces a person’s drift rate (Ratcliff et al., 2016). At the same time, when people engage in work, they may feel more time pressure and may be more likely distracted from the task of completing an EMA prompt. Environmental distractions and time pressures are important influences of careless responding (Meade & Craig, 2012) and lower response caution (Ratcliff et al., 2016), so that working activities should be associated with a lower boundary separation. Thus, we expected that working yields a temporarily lower drift rate and a lower boundary separation within a given person, resulting in weak or null effects of working on observed RTs. Conversely, we expected that when people are engaged in activities involving relaxing or regenerating, there would be a higher drift rate and a higher boundary separation within a person, again resulting in weak or null effects when inspecting observed RTs.

#### Hypothesis 4

At the within-person level, working is associated with lower drift rates (indirectly leading to slower RTs) and lower boundary separation (indirectly leading to faster RTs), whereas recreation is associated with higher drift rates (indirectly leading to faster RTs) and higher boundary separation (indirectly leading to slower RTs) for EMA affect ratings.

On the between-person level, we considered the effects of relatively stable person characteristics, namely, respondents’ neuroticism and depression levels, on affective information processing in EMA. Higher neuroticism and depression have been associated with slower processing of affective information (i.e., lower emotional clarity; Thompson et al., 2015) and slower information processing in general (Sočan & Bucik, 1998), suggesting lower drift rates for these individuals. With regards to boundary separation, higher levels of neuroticism and depression have been associated with a greater tendency for survey satisficing and careless responding (Conijn et al., 2020), and people with higher neuroticism have been shown to be more likely to adopt response strategies emphasizing speed over accuracy (Flehmig et al., 2010). Thus, past research suggests that higher neuroticism and depression levels should be associated with lower drift rates and lower boundary separation. We again expected that these effects would be conflated and weakened when examining observed RTs at face value.

#### Hypothesis 5

At the between-person level, higher neuroticism and depression are associated with lower drift rates (indirectly leading to slower RTs) and lower boundary separation (indirectly leading to faster RTs) for EMA affect ratings.

The final aim was to expand on prior research showing that observed RTs tend to decrease on average over the course of an EMA study and with repeated administration of the same momentary items (Arslan et al., 2021). To shed light on potential reactivity and practice effects in EMA, it would be valuable to know whether repeatedly completing EMA affect items changes how *fast* people can access and process affective information (drift rate), how *thoroughly* they access this information and how cautiously they are in responding (boundary separation), or both. Our preliminary position was that changes in RTs in an EMA study would in part reflect changes in both processes. In line with prior work suggesting that respondents may be less careful over time in EMA studies and may engage more in low effort responding (Eisele et al., 2022), we hypothesized that people’s boundary separation (response caution) would decrease over time. Whether repeatedly completing EMA affect items changes the accessibility of emotional information is less clear. Self-reported levels of emotional awareness have not been found to increase over time in EMA studies (Versluis et al., 2021). However, experiments on priming effects in attitude research suggest that concepts that an individual has thought about recently or thinks about frequently tend to be more easily retrieved than other concepts (Fazio, 1990); accordingly, we hypothesized that drift rates in EMA affect items would increase over time.

#### Hypothesis 6

Decreases in observed RTs for EMA affect ratings over the course of a study will be reflected in increasing drift rates and decreasing boundary separation.

## Method

### Participants and procedures

Data analyzed in this study were drawn from panel members in the Understanding America Study (UAS) who participated in EMA studies. The UAS is a probability-based Internet panel study managed by the Center of Economic and Social Research at the University of Southern California (Alattar et al., 2018). It currently has approximately 9000 members who were recruited through nationwide address-based sampling to ensure they were nationally representative. If and when sampled participants consent to becoming panel members, they are invited to participate in surveys on a regular basis until they choose to withdraw from the panel, or cease completing surveys. They are also sent information about various UAS affiliated sub-studies that they have the option to participate in.

The data were from a UAS sub-study conducted to examine momentary wellbeing experiences among participants aged 50 years and older. UAS panel members were eligible to participate if they were at least 50 years of age and if they used iOS or Android smartphones. The EMA study consisted of multiple measurement “bursts” (i.e., waves) with identical EMA items. A small portion of the EMA study bursts had the additional inclusion criteria of no prior participation in studies involving accelerometer use. Panel members meeting the inclusion criteria were sent information about the EMA study, and asked if they were willing to participate. Only a randomly sampled subset of eligible panel members that consented to participate were chosen to complete EMA data collection. For analyses in this study, if participants took part in more than one EMA study burst, only their first burst data were used.

Each burst involved completion of up to six EMA surveys daily, for one week, on a mobile app that participants installed on their personal smartphones. The app was programmed with the software NubiS (https://cesr.usc.edu/nubis/), a secure data collection and storage system created by the Center of Economic and Social Research at USC. Prior to the start of data collection, participants could select times for the first and last prompts for each day. The allowable time range for first prompts was between 6 and 11 AM, and for last prompts was between 7 and 11 PM. The times of surveys between the first and last prompts were randomly selected such that surveys were separated by time periods ranging from approximately one to three hours. After receiving a phone notification indicating it was time for a survey, participants had eight minutes to begin answering questions. Reminder alarms were sent if participants did not yet begin surveys four minutes after the initial notification. Each momentary survey took about two minutes to complete (including momentary questions not analyzed here). Apart from the EMA surveys, participants were also asked to complete end-of-day surveys and other assessments not analyzed in this study. For a subset of the EMA bursts, participants were also asked to wear an activity monitor over the study period (not analyzed here). Participants were compensated for completion of study procedures, which were approved by the local Institutional Review Board.

### Measures

#### EMA items and response times used in diffusion models

##### EMA negative affect items

Five items addressing momentary negative affect were administered at each EMA prompt. Participants were asked to rate how angry, dejected, frustrated, lonely, and stressed they felt before the prompt. All items were rated on a 0 to 100 horizontal visual analog scale with anchors that ranged from not at all to extreme.

##### EMA stressful events items

Three items about the experience of stressful events right before the prompt were analyzed to examine the correspondence of the drift–diffusion parameters derived from different item sets (i.e., negative affect and stressful events). One item asked if any stressful event occurred before the prompt, defined as any occurrence (even minor ones) that negatively affected the participant. The second item asked if participants were worrying about money, and the third item asked if they had experienced an argument with a significant other, spouse, close friend, or family member before the prompt. All three items were rated on a binary (yes/no) scale.

Several additional momentary questions were assessed in the EMA study but not used in the present analyses; these were pain intensity, happiness, fatigue, relaxation, cheerfulness, momentary location (“where were you”; 7 discrete choices) and social environment (“who were you interacting with”; 8 discrete choices). These EMA questions were not examined because the diffusion IRT model requires a set of items addressing one common (unidimensional) construct (see below), precluding the use of RTs for questions assessing multiple heterogeneous contents.

##### Response times

RTs for each item were recorded in the NubiS software used for item administration. The software recorded RTs for each individual question screen in integer seconds, where the time passed was counted from the moment at which NubiS sent a question screen to the browser to the moment it received a signal that the respondent had exited the screen. The recording of RTs included the time a respondent spent on the screen, but excluded any time on the server for processing answers and creating the next question screen.

#### Work and recovery activities

Momentary work and recovery activities were coded from one multicategory checklist (“check all that apply”) item included in each EMA prompt. Response options for the activity item were work, chores, leisure, inactive, interact with others, eating, drinking, on the telephone, and other. If work was reported, this was coded as 1 (yes) for the momentary engagement in work variable, and as 0 otherwise. Reporting leisure, inactive, interact with others, or on the telephone was coded as 1 (yes) for participation in recovery activities, and 0 otherwise. We assumed that “inactive” was a low activation state similar to relaxation, and that “interact with others” and “on the telephone” were comparable to socializing, consistent with a prior categorization scheme for recovery activities (Hernandez et al., 2021).

#### Neuroticism and depression

Questionnaires assessing neuroticism and depressive symptoms were administered repeatedly in 2-year intervals in the UAS. The questionnaires have been administered since 2016 for a total of up to 4 waves per person. Because respondents entered the UAS at different time points (the panel is still growing), the timing and number of the questionnaires differs across respondents. The scores included in the analyses were taken from the assessment an individual had completed closest in time before or after they completed the EMA burst. To limit potential biases resulting from nonconcurrent assessments, we only included assessments that had been completed within one year of the EMA study in the analyses; out of 954 participants in the EMA study, 876 (91.8%) had neuroticism and 852 (89.3%) had depression scores within one year. The average time difference (absolute number of days between questionnaire completion and EMA data collection) was 152 (*SD* = 97) days for neuroticism and 166 (*SD* = 103) days for depression.

Neuroticism was assessed with 8 items included in the 44-item Big Five Inventory (John & Srivastava, 1999). For each item, participants were asked to rate the extent to which they agreed that the given descriptor accurately characterizes them, on a scale of 1 (strongly disagree) to 5 (strongly agree). The Big Five Inventory has been widely used for personality research and has demonstrated good psychometric properties. Cronbach alpha for the neuroticism scale was 0.84 in the present sample.

Depressive symptoms were measured with an abbreviated 8-item version of the Center for Epidemiologic Studies Depression (CES-D) scale (Radloff, 1977). The CES-D is a widely used screening measure of depressive symptoms in the general population. It is intended to capture a continuum of psychological distress in the past week, with higher scores indicating greater depression symptom severity. The UAS administers an abbreviated 8-item version of the CES-D with binary response format, where participants were asked if statements were true or not for them. This 8-item version has demonstrated adequate construct validity (Steffick, 2000). Cronbach alpha was 0.86 in this sample.

### Statistical analysis

#### Diffusion model estimation

Many algorithms have been developed to estimate variants of the drift diffusion model. In the present study, we applied an item response theory (IRT) version of the model that was specifically designed for the analysis of subjective self-report ratings (Tuerlinckx & De Boeck, 2005; Tuerlinckx et al., 2016). The diffusion IRT model for self-report ratings is called D-diffusion model and it estimates drift rate and boundary separation as latent variables from binary responses and RTs across multiple items in a self-report measure. In contrast to diffusion models appropriate for cognitive ability tests, where decision making is expected to be fastest for individuals with the highest ability, the D-diffusion model assumes that responses are fastest for individuals at the extremes of the self-report construct. That is, people are expected to have less difficulty deciding on their answers (and, thus, faster RTs) when they have very high or very low negative affect levels compared to moderate affect levels. This “distance-difficulty” principle aligns with empirical results showing an inverted U-shaped relationship between RTs and affect ratings (Arndt et al., 2018).

As is typical for IRT, the model decomposes the model parameters into item- and person-parameters. This has the advantage that people’s drift rate and boundary separation parameters are adjusted for the fact that different self-report items are not completely interchangeable even if they commonly assess the same construct. That is, the diffusion IRT model acknowledges that different affect items vary in their respective “difficulties”, in contrast to traditional diffusion models in cognitive psychology experiments that often involve many interchangeable trials (Tuerlinckx & De Boeck, 2005; Tuerlinckx et al., 2016). An additional advantage is that this makes it possible to examine the psychometric fit of IRT diffusion models to the data (see below).

Specifically, the traditional diffusion model assumes that for a given person p and item h, a response $x_{ph}$ with response time $t_{ph}$ results from the following joint distribution function:

(1) $$h(x_{ph},t_{ph})=\frac{\pi }{\alpha^{2}}exp\left[\alpha \mu (x_{ph}-0.5)-\frac{\mu^{2}}{2}(t_{ph}-Ter)\right]\;\;\times \sum_{k=1}^{\infty }k\;sin(0.5\pi k)\;exp\left[-\frac{\pi^{2}k^{2}}{2\alpha^{2}}(t_{ph}-Ter)\right],$$

where μ is the drift rate, α the boundary separation, and Ter the nondecision time of a person. Whereas this traditional model treats the items as interchangeable, the IRT formulation of the D-diffusion model distinguishes person- and item-parameters for the drift rate and boundary separation with the following functions:

(2) $$\mu_{ph}=\theta_{p}-v_{h},\;\;\alpha_{ph}=\frac{\gamma_{p}}{a_{h}}$$

Here, the drift rate $\mu_{ph}$ is expressed as the difference between the person’s drift rate $\theta_{p}$ and item drift rate $v_{h}$ (the “difficulty” level of the item), and the boundary separation is given as the ratio of the person’s boundary separation $\gamma_{p}$ (the person’s “response caution”) and the item’s boundary separation $a_{h}$ (the “time pressure” of the item) (Tuerlinckx & De Boeck, 2005). Combining Eqs. 1 and 2, the probability of choosing the first response option on an item given a person’s drift rate and boundary separation is estimated using the following IRT D-diffusion model equation:

(3) $$P\left(x_{ph}=1|\theta_{p},\gamma_{p}\right)=\frac{exp\left[\frac{\gamma_{p}}{a_{h}}\left(\theta_{p}-v_{h}\right)\right]}{1+exp\left[\frac{\gamma_{p}}{a_{h}}\left(\theta_{p}-v_{h}\right)\right]}$$

We used the diffIRT package in R (Molenaar et al., 2015) to estimate the D-diffusion IRT model from the responses and RTs for the 5 negative affect items assessed at each EMA measurement occasion. The R code used to estimate the model is provided at https://osf.io/r82hn/. Because the model requires binary response data, we dichotomized the continuous affect ratings at the midpoint of the 0–100 scale (i.e., ratings from 0 to 49 were coded as 0, and ratings from 50 to 100 were coded as 1). A separate model was estimated for the 3 stressful events items; no recoding was necessary for the stressful events items because they were administered using a binary (yes/no) response format. Following prior research (Jaso et al., 2022), we excluded items for which the RTs were longer than 30 s (approximately 1% of the RT data) from the analyses. Furthermore, EMA measurement occasions with no variation in RTs were excluded because estimation of the diffusion models requires variation in RTs. Of 29,915 occasions, 848 (3%) were removed because of lack of variation in RTs.

After fitting the diffusion IRT model to the data, we derived factor score estimates of the drift rate and boundary separation parameters for each individual and EMA measurement occasion. The program estimates drift rates as the signed deviation from the item difficulties, such that drifts toward the upper boundary (affirmative responses) are positive and toward lower the boundary (non-affirmative responses) negative; because we were solely interested in the speed of information accumulation regardless of people’s response tendencies, we calculated absolute drift rates as the mean absolute (rather than signed) deviation of the person drift parameters from the item difficulties. Resulting drift rate and boundary separation factor scores were log transformed to normalize their distributions.

In addition to the diffusion parameters, we calculated a summary measure of the observed average RTs for each person and EMA measurement occasion. To compute this traditional RT measure, we log-transformed RTs of each negative affect item (excluding items for which the RTs were longer than 30 s), and then took the mean of the log-transformed RTs.

#### Preliminary psychometric analyses of diffusion model fit

We evaluated the fit of the diffusion IRT model to the data to ensure that the drift rate and boundary separation parameters were derived from a psychometrically sound measurement model. Following Tuerlinckx et al. (2016), we evaluated model fit separately for the item responses and for the RTs predicted by the IRT model. Regarding model fit for item responses, a fundamental assumption of IRT, including the diffusion IRT model, is that the items are indicators of a single dimension (essential unidimensionality). Thus, we estimated the fit of a unidimensional model for the affect response variables using confirmatory factor analysis (CFA) for binary outcomes. The CFA was conducted in M*plus* version 8.7 (Muthén & Muthén, 2017) using the WLSMV estimator, and employing cluster-robust standard errors to appropriately adjust the model fit values for the nesting of multiple EMA measurement occasions within individuals. To evaluate the fit for the model-implied RTs, we descriptively compared the observed and model-predicted RT distributions using histograms and quantile–quantile (Q-Q) plots (Tuerlinckx et al., 2016). For Q-Q plots, the quantiles of the RT distribution were plotted against the model-predicted quantiles, where the two are expected to be on a straight line if the diffusion model fits the data well.

#### Analysis of test–retest stability

To evaluate the test–retest reliability of the diffusion model parameters (and of the observed RT measure) across EMA measurement occasions, we estimated multilevel “null” models (i.e., multilevel models without a predictor). In this model, a given variable y (drift rate, boundary separation, and observed RTs) measured at time i for individual j is expressed as a linear combination of a grand mean across all people δ, between-person deviations $U_{i}$ from that grand mean, and occasion-specific within-person deviations $e_{ij}$ from each individual’s mean:

(4) $$y_{ij}=\delta +u_{j}+e_{ij},where\;u_{j}∼N(0,\tau^{2})\;and\;e_{ij}∼N(0,\sigma^{2})$$

The model has two variance components, one representing the variation in between-person means ($\tau^{2}$) and one representing occasion-specific variation within people ($\sigma^{2}$). From these variance components, we calculated the intraclass correlation (ICC) for each variable as a measure of test–retest stability:

(5) $$ICC=\frac{\tau^{2}}{\tau^{2}+\sigma^{2}}$$

The ICC is a measure of the average correlation between any two (randomly selected) measurement occasions.

#### Analysis of relationships between diffusion model parameters and observed RTs

Multilevel regression models were used to examine the relationships between the diffusion model parameters and observed RTs. At both the within- and between-person level, RTs were simultaneously regressed on drift rate and boundary separation parameters to examine the unique effects (standardized regression coefficients) and the total variance explained (squared multiple correlation) at each level.

We used a latent covariate approach (Lüdtke et al., 2008) implemented in M*plus* version 8.7 for this purpose. A common strategy in multilevel regression applications in which predictor variables (in this case, drift rate and boundary separation scores) are measured at each measurement occasion is to compute the mean of the observed scores across measurement occasions; the computed person-level mean and within-person centered deviations from this computed mean are then used as predictor variables to estimate between- and within-person effects of the independent variable in the regression model. This strategy can yield biased regression parameters because the computed (i.e., manifest) person-level mean is falsely assumed to be measured with perfect reliability (Lüdtke et al., 2008). The latent covariate approach has been shown to overcome this bias by using latent (rather than manifest) person means of the drift rate and boundary separation values as predictors of RTs at the between-person level (Lüdtke et al., 2008). The approach is based on a multivariate multilevel model in which all variables are decomposed into latent within- and between-person components. For RTs as the dependent variable y and for two independent variables (drift rate x and boundary separation z), each measured at occasion i for person j, the decompositions can be expressed as:

(6) $$y_{ij}=\delta_{y}+U_{yj}+R_{yij}\;x_{ij}=\delta_{x}+U_{xj}+R_{xij}\;z_{ij}=\delta_{z}+U_{zj}+R_{zij}$$

For each variable, δ is the grand mean across all people, U represents the latent between-person deviations from the grand mean, and R represents occasion-specific within-person deviations. In the multilevel regression model, the multilevel components of the drift-rate and boundary separation variables served as latent predictors at the within- and between-person levels:

(7) $$R_{yij}=\beta_{x,\text{within}}R_{xij}+\beta_{z,\text{within}}R_{zij}+e_{ij}\;U_{yj}=\beta_{x,\text{between}}U_{xj}+\beta_{z,\text{between}}U_{zj}+u_{j}$$

#### Correspondence of drift diffusion parameters across negative affect and stressful events items

To examine the correspondence of drift rate and boundary separation parameters derived from negative affect and stressful events items, we estimated the within- and between-person correlations between the diffusion model parameters across the two sets of items in multilevel models. To determine if the correlations of the same diffusion parameter between the two sets of items significantly exceeded the remaining correlations (i.e., the correlations between drift rate and boundary separation derived from the same set of items or from different sets of items), correlations were first Fisher z-transformed so that the sampling distribution of the correlations coefficients would be more normally distributed (Asuero et al., 2006). Significance tests were then conducted by testing the difference between dependent (i.e., correlated) correlations at the within- and between-person level of the multilevel model.

#### Construct validity analysis

Multilevel mediation models were used to analyze whether drift rate and boundary separation parameters showed the hypothesized competing indirect effects (i.e., indirect effects in opposite directions) when examining the relationship between the predictor variables and observed RTs. Four separate mediation models were estimated, one for each predictor variable: work (vs. other) activities and recovery (vs. other) activities served as binary predictors in separate within-person (so-called 1–1-1) mediation models, and neuroticism and depression served as continuous predictors in separate between-person (2–1-1) mediation models. In each model, drift rate and boundary separation were entered as simultaneous (i.e., multiple) mediators, and the observed RTs served as dependent variable. The mediation models were estimated with multilevel structural equation models (Preacher et al., 2010). The significance of indirect effects was determined with the product of coefficients method using Bayesian parameter estimation with default uninformative priors in M*plus* version 8.7. Like bootstrapping, 95% credible intervals derived from Bayesian estimation appropriately take the nonnormal distribution of indirect effects into account.

#### Analysis of change with repeated EMA administration

To investigate patterns of changes in observed RTs and in the diffusion model parameters over the course of the EMA study, we first descriptively inspected the sample means of each variable per EMA measurement occasion. Longitudinal changes in RTs in intensive longitudinal studies are often well described by a negative exponential function (Sliwinski et al., 2010). Accordingly, we fitted negative exponential growth curve models to each variable to formalize and compare the changes in the observed RTs and diffusion model parameters. These latent growth curve models assume a nonlinear pattern of change in a variable where rates of change (e.g., gains through practice) are most pronounced initially and then gradually slow down, with the variable approaching an asymptote. For a given variable y (i.e., RTs, drift rate, or boundary separation), measured at timepoint i for individual j, the model can be described as

(8) $$y_{ij}=a_{j}+g_{i}exp\left[-r_{j}\left(occasion_{ij}\right)\right]+e_{ij}$$

Here, $a_{j}$ refers to the person’s asymptotic level that would be reached with unlimited repeated measurement occasions, $g_{j}$ refers to “gain” as the overall amount of change from the person’s initial level to the final asymptote, and $r_{j}$ governs the rate of change (how quickly the curve moves toward its asymptote). We estimated the model as structural equation model, using M*plus* code developed by Preacher and Hancock (2015).

## Results

Data from a total of 954 participants were analyzed. Participants completed 29,067 out of 40,068 scheduled EMA measurement occasions. The median EMA completion rate was 75%, with an interquartile range of 28%. Descriptive characteristics of the sample are shown in Table 2. The sample was primarily female (62%), White (84%), and had an average age of 61.3 years (*SD* = 7.8). About one third of the sample reported annual household incomes of < $50,000 (30% of participants), $50,000-$99,999 (24%), and ≥ $100,000 (38%), respectively. The most frequently reported employment statuses were “currently working” (49%) and “retired” (28%).

### Fit of the diffusion IRT model

**Fig. 2:**
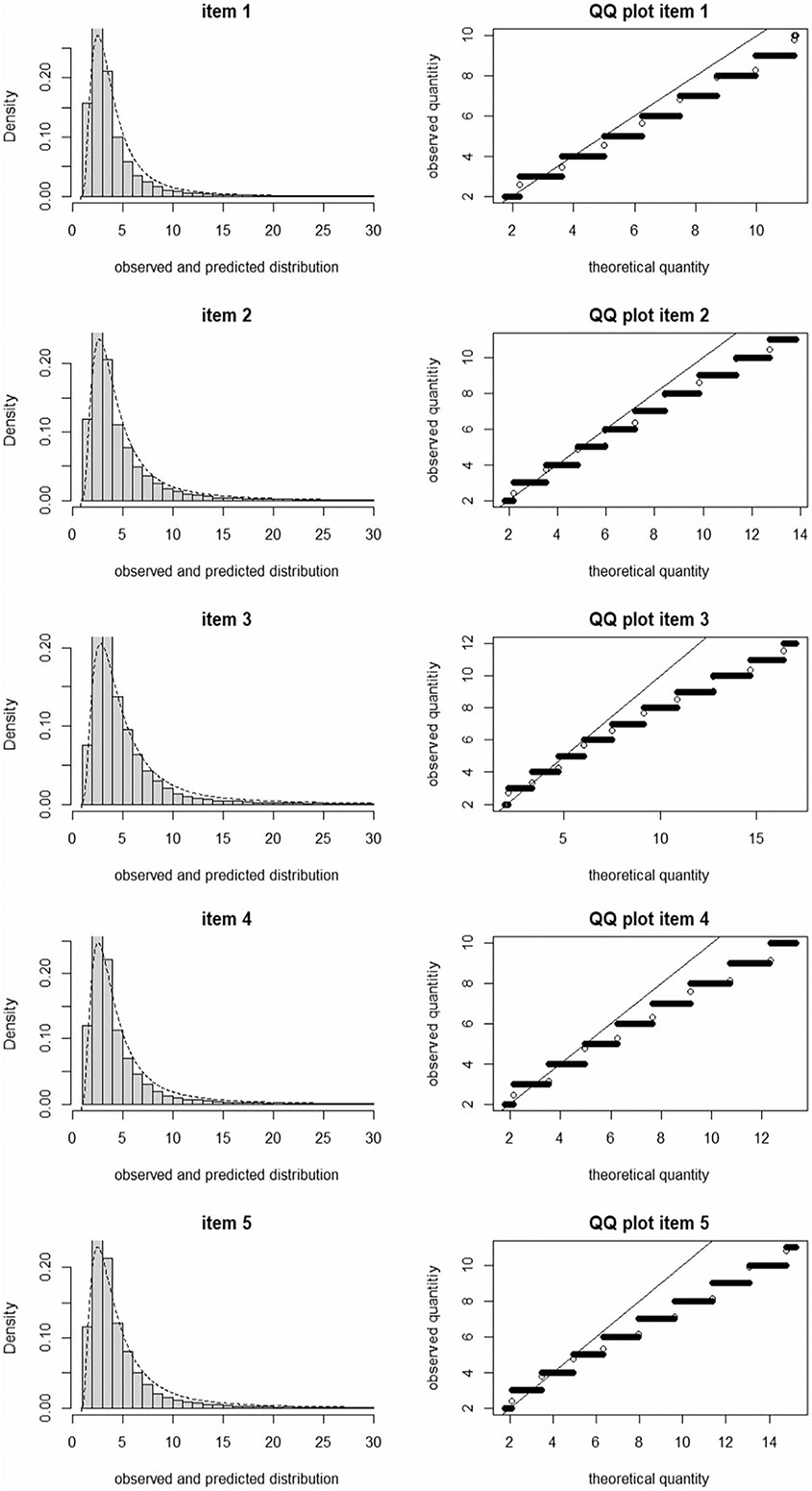
Histogram with corresponding QQ-plot of the predicted and observed response time distributions for the D-diffusion model applied to the five negative affect items. Item 1 = angry, item 2 = dejected, item 3 = frustrated, item 4 = lonely, item 5 = stressed

Confirmatory factor analyses supported a unidimensional model of the responses for the negative affect EMA questions. The following fit metrics were found for a single factor model with cluster robust standard errors: Global goodness of fit χ^2^(5) = 139.41, *p* < 0.001; CFI = 0.989; TLI = 0.978; RMSEA = 0.030 (90% CI = 0.026—0.035); SRMR = 0.060. Examining the fit of the diffusion IRT model to implied RTs using histograms and Q-Q plots (Fig. 2), we found that the observed and predicted RT distributions closely coincided for all of the negative affect items, indicating an overall good fit, even though the Q-Q plots showed some apparent misfit at the upper end (i.e., for longer RTs) where fewer observations were present (Molenaar et al., 2015).

**Fig. 3:**
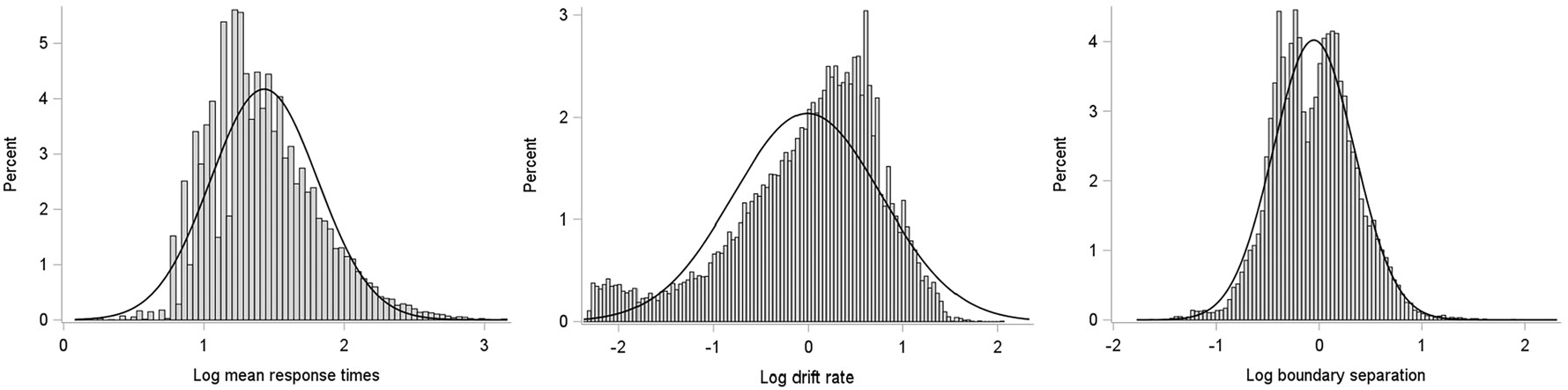
Distributions of log response times, log drift rate, and log boundary separation across all EMA measurements, with normal density curve

Inspecting the factor scores of drift rates and boundary separation derived from the model, the log-transformed boundary separation showed outliers with extreme negative values for 5 (< 0.0001%) EMA measurement occasions; these observations were set to missing. The resulting log-transformed observed RT, drift rate, and boundary separation variables were approximately normally distributed (see Fig. 3).

### Hypothesis 1: Test–retest stability

Table 3 shows the means, within- and between-person variance components, and ICC for each variable. The observed RTs showed moderate test–retest stability, ICC = 0.408 (*SE* = 0.016, *p* < 0.001). Test–retest stabilities for the diffusion parameters were also moderate in magnitude, with ICC = 0.312 (*SE* = 0.013, *p* < 0.001) for drift rates and ICC = 0.390 (*SE* = 0.013, *p* < 0.001) for boundary separation values.

### Hypothesis 2: Relationships of drift rate and boundary separation with observed RTs

At the within-person level, greater momentary drift rates predicted smaller (i.e., faster) observed RTs (β=-0.690, *SE* = 0.002, *p* < 0.001) and greater momentary boundary separation predicted larger (i.e., slower) RTs (β=0.610, *SE* = 0.003, *p* < 0.001) in multilevel multiple regression analysis, together accounting for 85% of the within-person variance in RTs. Similarly, at the between-person level, greater person-mean drift rates predicted faster RTs (β=-0.630, *SE* = 0.015, *p* < 0.001) and greater boundary separation predicted slower RTs (β=0.753, *SE* = 0.013, *p* < 0.001), together accounting for 97% of the between-person variance in RTs.^1^

### Hypothesis 3: Correspondence across negative affect and stressful events items

Within- and between-person correlations among the diffusion parameters calculated from the negative affect and stressful events items are shown in Table 4. Corresponding diffusion parameters were positively correlated with each other across the different sets of EMA items (*r*s = 0.37 to 0.39 within-person, *r*s = 0.66 to 0.77 between-person; *p*s < 0.001). Furthermore, these correlation coefficients were all significantly (*p*s < 0.001) greater in absolute magnitude compared to the correlations between non-corresponding diffusion parameters (i.e., drift rate with boundary separation within and across sets of items) at the same analysis level (*r*s ranging from −0.08 to −0.31 within-person, *r*s ranging from −0.02 to −0.30 between-person).

### Construct validity

#### Hypothesis 4: Within-person mediation - work and recovery activities

**Fig. 4:**
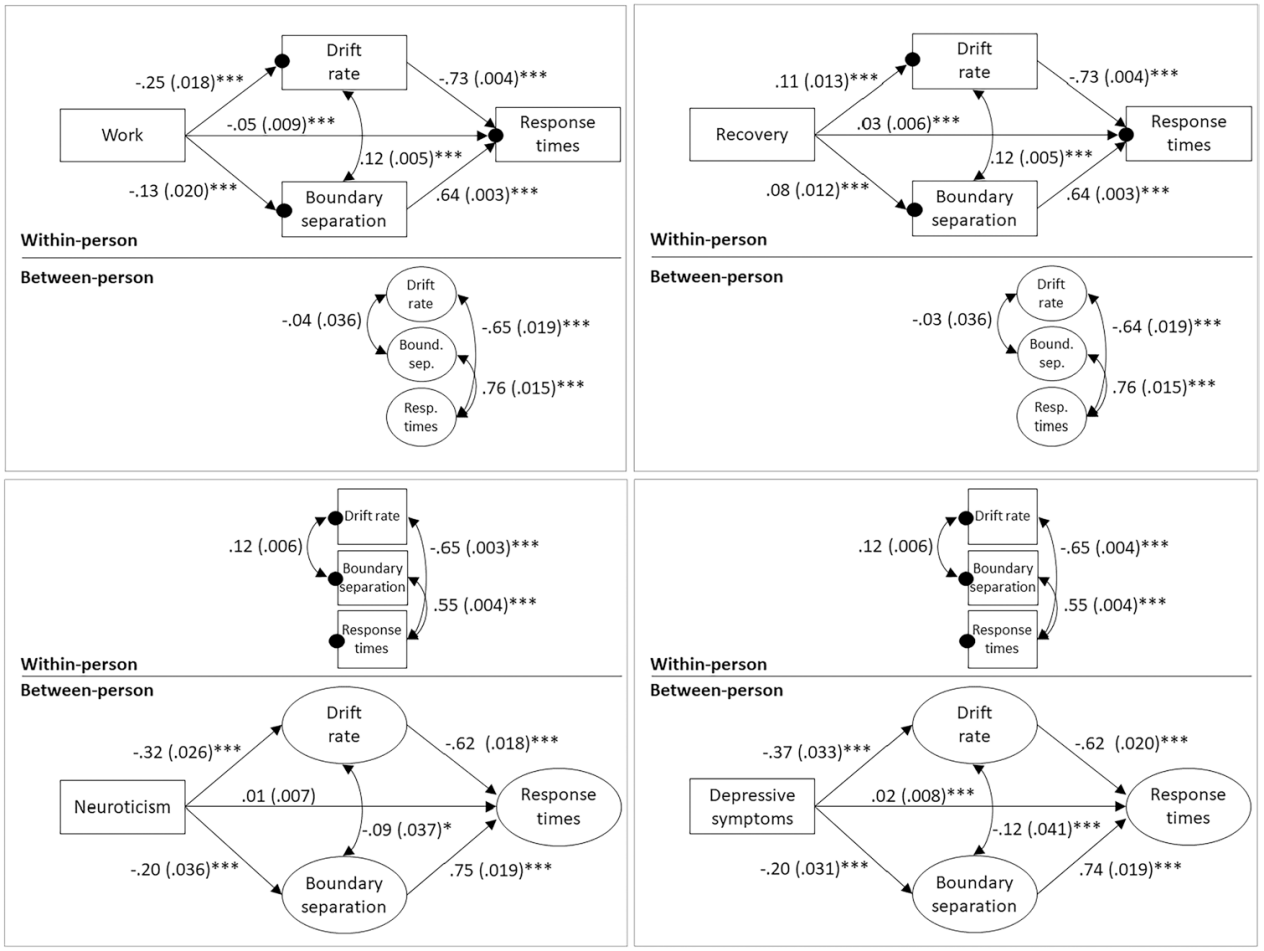
Results of multilevel mediation models examining within-person effects of work and recovery activities (top panel) and between-person effects of neuroticism and depressive symptoms (bottom panel) on log response times via log drift rate and log boundary separation. Variables in boxes represent observed variables. Small filled circles on the within-person level represent random intercepts, which are shown as circles representing latent variables on the between-person level. Single headed arrows indicate regression (path) coefficients, and double headed arrows connect variables that are allowed to correlate with each other. Standardized regression and correlation coefficients are shown. Because work and recovery activities are dichotomous variables, coefficients involving these two variables as predictors are only standardized with respect to the dependent variable. For all other variables, coefficients are standardized with respect to the independent and dependent variable. Values in parentheses are standard errors. * *p* < .05; *** *p* < .001

Engaging in work showed a significant positive total effect on (i.e., bivariate association with) observed RTs, but the association was of small magnitude (β=0.054, *SE* = 0.017, *p* < 0.001). As shown in Fig. 4 (top left panel), in the mediation model, engaging in work was associated with lower momentary drift rates (β=-0.254, *SE* = 0.018, *p* < 0.001), and lower drift rates in turn predicted slower RTs (β=-0.732, *SE* = 0.004, *p* < 0.001). Conversely, work was associated with a lower momentary boundary separation (β=-0.133, *SE* = 0.020, *p* < 0.001), and a lower boundary separation in turn predicted faster RTs (β=0.638, *SE* = 0.003, *p* < 0.001). As hypothesized, indirect effects for the relationship between working and observed RTs were significant and in opposite directions for drift rates (indirect effect = 0.054, 95% CI = 0.047 to 0.064, *p* < 0.001) and boundary separation (indirect effect = −0.025, 95% CI = −0.033 to −0.018, *p* < 0.001). The direct effect of work on observed RTs (i.e., after accounting for the effects of drift rate and boundary separation) was significantly positive yet small (β=-0.047, *SE* = 0.009, *p* < 0.001).

Recovery (versus other activities) did not show a significant total effect on momentary observed RTs (β=0.003, *SE* = 0.012, *p* = 0.901). In the mediation model (Fig. 4, top right panel), recovery was associated with a significantly higher momentary drift rate (β=0.106, *SE* = 0.013, *p* < 0.001), which predicted faster RTs (β=-0.730, *SE* = 0.004, *p* < 0.001), and recovery was associated with a significantly higher boundary separation (β=0.075, *SE* = 0.012, *p* < 0.001), which predicted slower RTs (β=0.637, *SE* = 0.003, *p* < 0.001). Indirect effects were significant and in opposite directions for drift rates (indirect effect = −0.023, 95% CI = −0.028 to −0.017, *p* < 0.001) and boundary separation (indirect effect = 0.014, 95% CI = 0.009 to 0.019, *p* < 0.001). The direct effect of recovery activities on observed RTs was significantly positive yet small (β=0.032, *SE* = 0.006, *p* < 0.001).

#### Hypothesis 5: Between-person mediation - neuroticism and depressive symptoms

On the between-person level, higher neuroticism levels did not show a significant total effect on a person’s average RTs (β=0.060, *SE* = 0.032, *p* = 0.061). In the mediation model (Fig. 4, bottom left panel), higher neuroticism was associated with a significantly lower average drift rate (β=-0.317, *SE* = 0.026, *p* < 0.001), which in turn predicted slower RTs (β=-0.622, *SE* = 0.018, *p* < 0.001), and with a significantly lower boundary separation (β=-0.197, *SE* = 0.036, *p* < 0.001), which predicted faster RTs (β=0.748, *SE* = 0.019, *p* < 0.001). Indirect effects of neuroticism on RTs were significant and in opposite directions for drift rate (indirect effect = 0.007, 95% CI = 0.006 to 0.009, *p* < 0.001) and boundary separation (indirect effect = −0.005, 95% CI = −0.008 to −0.004). The direct effect of neuroticism on observed RTs was not significant (β=0.010, *SE* = 0.007, *p* = 0.180).

Higher depressive symptom levels showed a significantly positive total effect on observed RTs, such that people with more depressive symptoms had slower RTs (β=0.104, *SE* = 0.031, *p* < 0.001). As shown in Fig. 4 (bottom right panel), higher depressive symptoms were associated with a lower drift rate (β=-0.371, *SE* = 0.033, *p* < 0.001), predicting slower RTs, and a lower boundary separation (β=-0.195, *SE* = 0.031, *p* < 0.001), predicting faster RTs. Indirect effects were significant and in opposite directions for drift rate (indirect effect = 0.026, 95% CI = 0.021 to 0.031, *p* < 0.001) and boundary separation (indirect effect = −0.017, 95% CI = −0.023 to −0.011, *p* < 0.001). The direct effect of depressive symptoms on observed RTs was significant yet small (β=0.021, *SE* = 0.008, *p* = < 0.001).

### Hypothesis 6: Change with repeated EMA administration

**Fig. 5:**
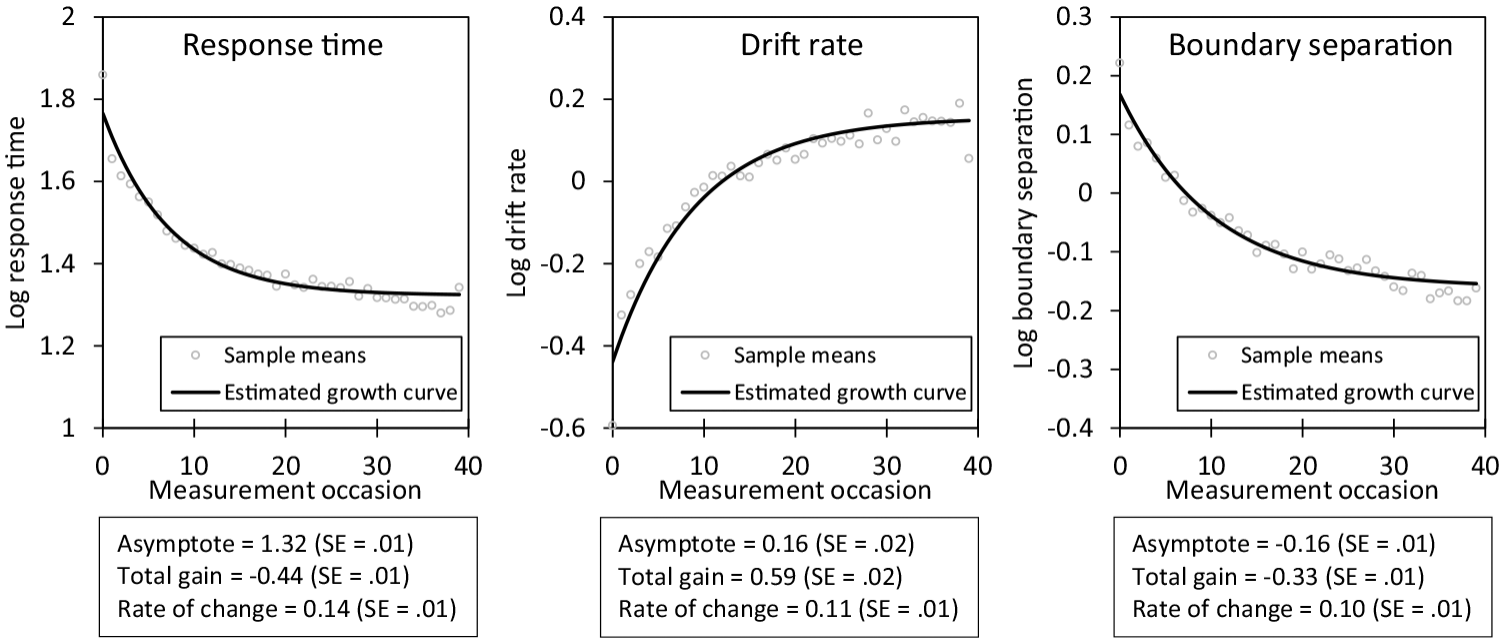
Changes in log response times, log drift rate, and log boundary separation over the course of the EMA study

Observed RTs for the EMA affect ratings decreased over the course of the study with repeated measurement occasions. As shown in Fig. 5, the mean observed RT at the first EMA occasion was 1.86 log seconds (back-transformed median = 6.42 s), and the observed RTs decreased to 1.40 log seconds (back-transformed median = 4.06 s) after 10 to 12 measurements. The pattern of change in RTs was well described by a negative exponential growth curve, χ^2^ (811) = 1403.65 (*p* < 0.001), CFI = 0.955, TLI = 0.957, RMSEA = 0.029 (90% CI = 0.027—0.031), SRMR = 0.047. The growth function showed a significant negative gain parameter (−0.442, *SE* = 0.009, *p* < 0.001) over time.

For the diffusion parameters, a negative exponential growth curve also fit the data reasonably well: for drift rates, χ^2^ (811) = 1534.74 (*p* < 0.001), CFI = 0.927, TLI = 0.930, RMSEA = 0.031 (90% CI = 0.028—0.033), SRMR = 0.050; and for boundary separation, χ^2^ (811) = 1170.41 (*p* < 0.001), CFI = 0.973, TLI = 0.974, RMSEA = 0.022 (90% CI = 0.019—0.024), SRMR = 0.036. As shown in Fig. 5, respondents’ drift rates significantly increased (gain parameter = 0.594, *SE* = 0.021, *p* < 0.001), whereas respondents’ boundary separation significantly decreased (gain parameter = −0.329, *SE* = 0.010, *p* < 0.001) over the course of the EMA study.

To examine whether the changes were more pronounced for people’s drift rate or boundary separation, we transformed the parameters into z-scores and compared the gain parameters in a multivariate negative exponential growth model with both parameters modeled simultaneously. The gains were −0.825 (*SE* = 0.025) z-scores for boundary separation and + 0.752 (*SE* = 0.026) z-scores for drift rates, suggesting pronounced changes of three-fourths of a standard deviation or more for both parameters. The changes (absolute magnitude of gain parameters) were marginally more pronounced for boundary separation compared to the drift rate (Wald-test χ^2^[1] = 3.06, *p* = 0.080).

## Discussion

The drift diffusion model is one of the most successful RT-based mental process models for understanding decision-making in experimental psychology. In this study, we examined whether the model could be successfully applied to RTs in EMA for the purpose of capturing and disentangling basic mental process components as people rate their momentary experiences in their everyday natural environments. Several results are noteworthy.

We found that both parameters from the diffusion model showed moderate stability across EMA measurement occasions (supporting Hypothesis 1), in magnitudes comparable (even though slightly lower than) the stability of observed RTs. Roughly 30–40% of the variance in drift rate and boundary separation values were attributable to stable between-person differences, and 60–70% to occasion-specific variation within people, suggesting that between- and within-person variance components in these mental process parameters can be reliably distinguished in EMA RT data. This result is significant especially in view of the fact that experimental research commonly administers dozens (or even hundreds) of decision tasks to apply cognitive process models such as the diffusion model (Ratcliff et al., 2016). By contrast, we estimated drift rate and boundary separation values from RTs for only 5 negative affect items at each EMA measurement occasion. Despite the modest number of affect items, we found moderate test–retest stability in the diffusion model parameters, suggesting that relatively stable individual differences in the parameters can be captured using brief EMA affect scales.

Supporting Hypothesis 2, drift rate and boundary separation variables explained the majority of the variance in the observed RTs, both within and between individuals, consistent with the argument that they represent separable mental process components underlying the observed RTs to EMA items. From a practical perspective, it is noteworthy that the diffusion model assumes a dichotomous decision process (Tuerlinckx et al., 2016), whereas EMA questions are most commonly administered using visual analog or ordinal response scales, each of which has numerous response options (May et al., 2018). In line with Hypothesis 3, we found that diffusion parameters derived from a set of visual analog items –for which we dichotomized the responses– showed moderate to high correlations with corresponding parameters derived from a separate set of EMA items that were administered in dichotomous response format, suggesting that differences in response scales may not heavily impact the results. This speaks to the possibility that the drift diffusion model may have wide applicability in EMA research.

Our primary (construct) validity question was whether the diffusion model parameters related in theoretically expected ways to within-person changes in activities (momentary work and recreation) and to person-level characteristics (neuroticism and depression), and whether these relationships were confounded when observed RTs are taken at face value. As hypothesized (Hypothesis 4), the activity of working was associated with momentarily lower drift rates and lower boundary separation, consistent with the idea that people are more likely distracted when they are engaged in work and thus have more difficulty to processing the information necessary to complete EMA items both quickly (drift rate) and thoroughly (boundary separation). The reverse pattern was evident when participants engaged in recreational activities. Furthermore, respondents with higher neuroticism and depression showed generally lower drift rates and lower boundary separation (Hypothesis 5), in line with the idea that mental health problems impede the ability to process affective information quickly and thoroughly. Importantly, when examining observed RTs, the effects of these predictor variables were much reduced (for work and depression) or even nonsignificant (for recreation and neuroticism), because the indirect effects through drift rates and boundary separation partially or fully canceled each other out. This highlights that extreme caution should be exercised when using observed RTs indiscriminately as proxy indicators of diverse processes such as emotional clarity (i.e., the ability to access affective information quickly) (Lischetzke et al., 2005) or survey satisficing.(i.e., cursory information processing) (Jaso et al., 2022) in EMA research, because relying on observed RTs alone are likely to yield spurious and misleading results that could be disambiguated by the drift diffusion model.

Finally, expanding on prior research documenting a decrease in observed RTs over the course of an EMA study (Arslan et al., 2021), we found that these decreases were reflected in increasing drift rates and decreasing boundary separation over the 7-day EMA protocol (Hypothesis 6). Our results are in line with previous findings by Dutilh et al. (2009), who applied a drift diffusion model to reaction time data from lexical decision tasks. They showed that repeated practice on the task led to increased drift rates and decreased boundary separation, suggesting that practice effects consist of multiple cognitive processing components. These results are interesting in light of prior research that has found little evidence for reactive effects of repeated EMA assessments when focusing on the self-reported levels of momentary experiences and behaviors (Cerino et al., 2022). Our findings suggest that even if levels of self-reported experiences are not affected, there are pronounced shifts in the cognitive processing of affective information with repeated momentary self-reporting.

### Limitations and future directions

This study has several limitations. Even though the sample was relatively large and drawn from a nationally representative panel, only participants who were 50 years and older were included in the study, and the results may not generalize to younger people. Participants completed the EMA surveys on their own smartphones, which may have introduced selection biases by excluding individuals who did not own a smartphone. The compliance rate with the EMA protocol was 75% and lower than the average compliance rate of 79% reported in a recent large meta-analysis of EMA studies (Wrzus & Neubauer, 2023), which may have further biased the results if skipped EMA reports were not missing at random. Furthermore, even though our results are in line with the idea that drift rate and boundary separation are underlying components of people’s RTs in EMA, the observational study design precludes causal interpretations.

Our results are limited to RTs for negative affect and may not generalize to other content domains examined with EMA. It is important to note, however, that our choice to focus on negative momentary affect was done for pragmatic and methodological reasons and does not suggest that future research should limit their analyses to this particular psychological dimension. Instead, we encourage future research to examine the drift diffusion model with a range of different momentary constructs.

Even though we found correspondence between diffusion parameters derived from responses on items that were presented in binary (yes/no) format and diffusion parameters resulting when responses on continuous rating scales were dichotomized, we note that our decision to dichotomize the responses along the scale midpoint was one of many possible options. We selected the scale midpoint as cutoff because midpoint responses on rating scales are often regarded as an expression of uncertainty or indifference, whereas responses above and below the midpoint are viewed as an expression of a choice for a direction of an opinion (Chyung et al., 2017; Shmueli, 2005). However, responses on continuous rating scales may involve multiple simultaneous or consecutive decisions. For example, Böckenholt (2012) proposed multiple sequential decision processes that give rise to respondents’ selection of a response, including the decision to express an opinion or not (e.g., choose the midpoint or not), the direction of an opinion (i.e., whether to choose a response above or below the scale midpoint) and the intensity of the attitude or experience (i.e., whether to choose a more or less extreme response). If multiple sequential decisions underly responses to continuous items, the diffusion model as a model developed for binary (yes/no) decisions may not provide an ideal representation of the mental processes involved in responding to these items.

As is typical for EMA studies, participants in this study were instructed to report their momentary experiences right before the prompt. However, recent research (e.g., Wen et al., 2021) has demonstrated that participants in EMA studies do not necessarily always adhere to the instructed time frame and that they may differ in the time period they draw upon when answering momentary questions (e.g., some participants may think of the moment right before the prompt, others may think of a longer time period of one or more hours before the prompt, others may think of their general experiences). As suggested by Robinson and Clore’s (2002) accessibility model of emotional self-report, the accessibility of emotional experiences may well vary with the length reporting period. Thus, if drift rates derived from the diffusion model are a sensitive measure of the accessibility of emotional information, it might be speculated that this diffusion parameter may potentially be useful as an indirect measure to gain information about the time period people use when responding to EMA items. Examining this possibility could be an interesting agenda for future research.

This study provides initial evidence that the drift diffusion model may enhance understanding of the response process to affective items in EMA, yet more work is needed to investigate the validity and interpretation of the diffusion model parameters in EMA contexts. Notably, the large majority of studies utilizing the model have collected data from cognitive decision-making tasks, where a person’s drift rate is often interpreted in terms of processing speed (i.e., cognitive ability) (Ratcliff et al., 2016). Applied to the task of momentary affective self-reporting, drift rates may best be viewed as the speed of accessing and processing affective information. That is, we speculate that they reflect a combination of the accessibility of emotions in particular (e.g., emotional clarity, attitude strength) and a person’s processing speed in general (i.e., cognitive functioning). The extent to which drift-rates derived from EMA items are domain-specific or generalize across experience domains could be addressed in future research by comparing drift rates across diverse EMA contents (e.g., affect, bodily sensations, physical symptoms, momentary thoughts and attitudes). Aspects of drift-rates that generalize across different EMA contents may theoretically be attributable to differences in general processing speed, which could be validated by correlating drift rates from diverse EMA contents with mobile measures of cognitive functioning (Sliwinski et al., 2018).

On the other hand, aspects of drift-rates that are specific to EMA contents may be attributable to the accessibility of particular experiences. For instance, when investigating interoception, drift rates may represent accessibility of bodily sensations. This could be addressed in a study where participants are asked EMA questions regarding how fast their heart rate feels, and heart rate is also recorded by a wearable device (Höller et al., 2021). The association between these two measures would be expected to be greater for individuals with a higher drift rate calculated from the heart rate items. This would provide initial evidence that the response times from bodily sensation items may be used to capture momentary interoception ability. Along the same lines, predictions for other experience domains captured with EMA could be tested, as well.

Similarly, as boundary separation is viewed as an expression of the speed-accuracy trade-off, additional validation is warranted to understand the implications of low boundary separation in terms of the quality of people’s EMA reports. Specifically, fast RTs have been viewed as an indicator of careless responding in EMA research in particular (Jaso et al., 2022) and in survey research in general (Curran, 2016; Schneider et al., 2018), potentially reducing the quality of self-reports. However, given that fast RTs may originate from high information accessibility (high drift rates) or low response caution and cursory information processing (low boundary separation), it may be speculated that low boundary separation is a more precise and valid indicator of careless responding compared to RTs taken at face value. In future research, this conjecture could be investigated by examining the extent to which boundary separation values correlate more strongly than observed RTs with other indicators of survey satisficing and careless responding derived from EMA response patterns, including measures of random responding, “straightlining”, or outlier responses (Curran, 2016; Meade & Craig, 2012). Examining boundary separation may also allow researchers to investigate fluctuations in the quality of EMA responding as a function of situational factors (e.g., momentary distractions) and the position of an item in the EMA survey (e.g., beginning versus the end). For example, in a study examining potential survey fatigue, items administered towards the end of EMA surveys may be expected to be associated with lower momentary boundary separation compared to those at the beginning, particularly if EMA surveys are lengthy.

## Conclusions

The present study demonstrates the feasibility of applying the drift diffusion model to RTs for momentary affect reports in EMA, and suggests that the model can reliably distinguish basic components of the mental processes underlying people’s responses to EMA questions. The rise of smartphone EMA data collection has made RTs readily available as an unobtrusive and cost-effective data source, which opens many opportunities for using the drift diffusion model to enhance our understanding of how people answer momentary questions about their experiences in daily life.

## Figures and Tables

**Table 1:** Differences between traditional applications of the drift diffusion model versus its application to Ecological Momentary Assessment

Characteristics	Typical drift diffusion model applications	Drift diffusion model application to EMA
Task	Simple decision tasks with objectively correct and incorrect answers	Self-reports of current emotions and experiences, no objectively correct answer
Number and type of test items	Dozens to hundreds of trials involving homogeneous test items	Few self-report questions addressing noninterchangeable contents
Response options	Binary choices	Varying response scale formats
Instructions	Typically, respondents are asked to be fast yet accurate	No explicit instructions regarding time limit
Setting	Controlled laboratory conditions	Everyday life contexts
Frequency of measurement	Single (or few) session(s)	Densely repeated measurement multiple times per day, across multiple days
Purpose of measurement	Investigation of person characteristics (presumably stable)	Ability to investigate within-person dynamic processes and stable between-person characteristics

**Table 2:** Participant characteristics

Characteristic	*n*	Mean (*SD*) or percentage
Age (years)	953	61.3 (7.8)
Gender		
Male	365	38%
Female	589	62%
Race		
White	799	84%
Black	61	6%
American Indian or Alaska Native	12	1%
Asian	27	3%
Hawaiian/Pacific Islander	5	1%
Mixed	46	5%
Hispanic		
Yes	79	8%
No	875	92%
Employment status		
Currently working	465	49%
On sick/other leave	2	0%
Unemployed	36	4%
Retired	266	28%
Disabled	60	6%
Other	124	13%
Education		
High school grad or less	142	15%
Some college, no degree	241	25%
Associate’s degree	141	15%
Bachelor’s degree	247	26%
Graduate degree	183	19%
Annual household income		
< $50,000	282	30%
$50,000-$99,999	231	24%
≥ $100,000	365	38%
Do not wish to provide	76	8%

**Table 3:** Means and variance components for log-transformed response times, drift rates, and boundary separation values

Variable	Mean	Variance		Intraclass correlation
		Between-person	Within-person	
Log response times	1.425	0.058	0.084	0.408
Log drift rates	−0.028	0.193	0.425	0.312
Log boundary separation	−0.048	0.062	0.096	0.390

**Table 4:** Between-person (above main diagonal) and within-person (below main diagonal) correlations between diffusion model parameters derived from the negative affect items and stressful events items

	Negative affect drift rate	Negative affect boundary separation	Stressful events drift rate	Stressful events boundary separation
Negative affect drift rate	-	−0.02, *p* = 0.827 (B)	**0.66,** *p* **< 0.001 (B)**	−0.28, *p* < 0.001 (B)
Negative affect boundary separation	0.11, *p* < 0.001 (W)	-	−0.26, *p* < 0.001 (B)	**0.77,** *p* **< 0.001 (B)**
Stressful events drift rate	**0.39,** *p* **< 0.001 (W)**	−0.08, *p* < 0.001 (W)	-	−0.30, *p* < 0.001 (B)
Stress boundary separation	−0.19, *p* < 0.001 (W)	**0.37,** *p* **< 0.001 (W)**	−0.31, *p* < 0.001 (W)	-

*B* Between-person correlation; *W* Within-person correlation. Correlations for variables addressing the same drift diffusion parameter are shown in bold font

## Data Availability

The datasets analyzed during the current study can be freely accessed at https://uasdata.usc.edu/index.php for registered users.

## Footnotes

1 Using the person-level averages of drift rate and boundary separation values calculated as manifest predictor variables in the multilevel model predicting RTs yielded results that were similar to those from the model using the latent covariate approach, with within-person regression coefficients of β=-.711 (*SE* = .002, *p* < .001) for drift rates and β=.663 (*SE* = .002, *p* < .001) for boundary separation, and between-person regression coefficients of β=-.636 (*SE* = .006, *p* < .001) for drift rates and β=.764 (*SE* = .004, *p* < .001) for boundary separation.

## Code availability

Software code is available at https://osf.io/r82hn/.
